# Metabolites as key signaling mediators: rethinking diabetic kidney disease from a network perspective

**DOI:** 10.3389/fendo.2026.1969901

**Published:** 2026-10-23

**Authors:** Xin Li, Xu Wang, Cheng Fang, Zhonghong Yan, Lei Liu, Jinwei Han, Liangyou Zhao, Xiuhong Wu

**Affiliations:** 1 State Key Laboratory of Integration and Innovation of Classic Formula and Modern Chinese Medicine, Heilongjiang University of Chinese Medicine, Harbin, China; 2 Qiqihar Medical University, Qiqihar, China; 3 Heilongjiang University of Chinese Medicine, Harbin, China

**Keywords:** biomarkers, diabetic kidney disease, kidney, metabolites, spatial metabolomics

## Abstract

Diabetic kidney disease (DKD) remains the leading cause of end-stage renal disease worldwide. Current diagnostic approaches rely mainly on albuminuria and eGFR, but these are insufficiently sensitive for detecting early lesions, and treatment options remain limited. Metabolites are not merely byproducts of metabolism but serve as central signaling hubs that orchestrate DKD progression. These metabolites can act as receptor ligands, substrates for epigenetic modifications, and cargoes of extracellular vesicles. Together, they form a complex metabolic signaling network that links systemic metabolic disturbances to local kidney injury. Adopting this metabolite-centric new perspective transforms DKD from a collection of isolated pathways into a systemic, network-level disease. Recent advances in multidimensional metabolomics technologies—including static profiling, spatial metabolomics, metabolic flux analysis, and multi-omics approaches—enable us to dissect the metabolic network of DKD with unprecedented resolution. This review synthesizes current knowledge on metabolite-driven mechanisms, including the AGEs–RAGE pathway, lipotoxicity, amino acid reprogramming, and EV-mediated crosstalk. It systematically categorizes metabolite biomarkers according to their clinical functions, covering early detection, progression prediction, and pathogenic drivers. In addition, emerging therapeutic strategies targeting specific metabolic nodes are discussed. Finally, building on these established findings, we propose a hypothetical roadmap toward metabolic precision medicine. This framework hypothesizes the stratification of patients into distinct metabolic phenotypes, such as the high-TMAO, high-BCAAs, and high-AGEs subtypes. It suggests that treatment regimens could be selected based on the underlying metabolic mechanisms and that therapeutic responses could be assessed by dynamic monitoring of circulating metabolites. If validated, this conceptual strategy holds promise for overcoming current diagnostic and therapeutic bottlenecks and improving outcomes for patients with DKD.

## 1 Introduction

Diabetic kidney disease (DKD) remains the leading cause of end-stage renal disease (ESRD) worldwide. Approximately 40% of patients with diabetes develop DKD (1). Although some progress has been made in glycemic control and blood pressure management, the residual risk of progression to renal failure remains high (2, 3). The main reason for this clinical dilemma is that clinical practice still relies on traditional biomarkers such as proteinuria and estimated glomerular filtration rate (eGFR). These markers only detect established kidney injury and fail to identify early metabolic disturbances (4, 5). Consequently, for many patients, kidney damage is already irreversible at the time of diagnosis. This underscores the urgent need to establish new research paradigms to achieve early detection of DKD and to implement mechanism-based interventions.

Historically, metabolites were regarded only as byproducts or intermediates of metabolic pathways. Modern studies have shown they are important signaling molecules that regulate cellular functions and intercellular communication (6). They can serve as ligands for membrane receptors and participate in signal transduction, for example, through the binding of advanced glycation end products (AGEs) to their receptors. They can also act as substrates or cofactors for epigenetic modifications, such as acetyl-CoA in histone acetylation and α-ketoglutarate in DNA demethylation. In addition, they mediate intercellular communication among different renal cells via extracellular vesicles (EVs) (7–10). Small-molecule metabolites have diverse functions and thus represent a key link connecting systemic metabolic disorders with local pathological changes in the kidney (**Figure 1**).

**Figure 1:**
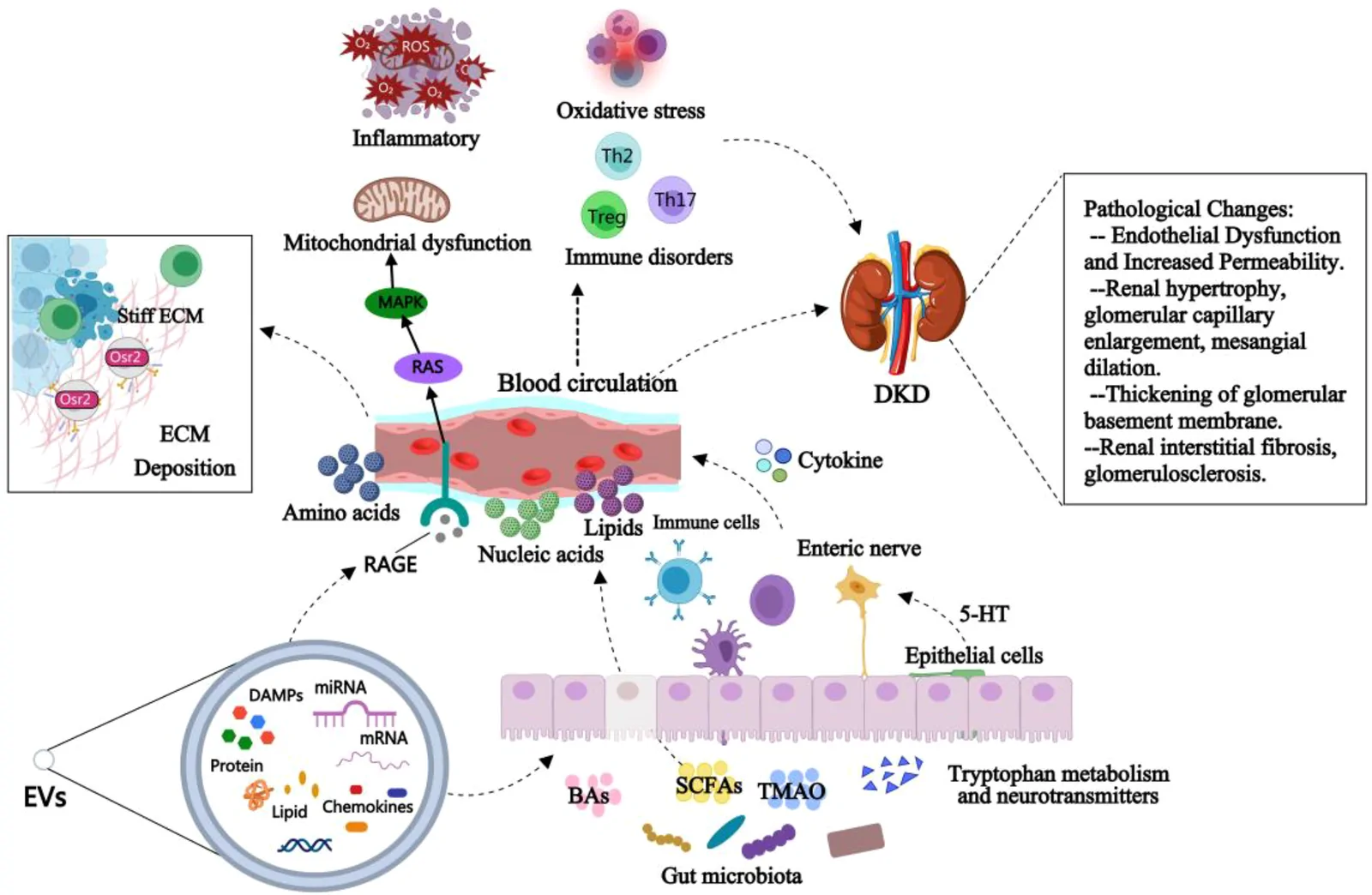
Role of metabolites in DKD. Extracellular AGEs bind to RAGE (receptor for AGEs) and activate MAPK signaling pathways, thereby promoting inflammatory responses in recipient cells. In parallel, EVs carry various biomolecules, including nucleic acids, proteins, lipids, DAMPs, cytokines, and chemokines. After EVs are taken up by recipient cells or interact with surface receptors, EV cargo can modulate recipient-cell signaling through RAGE-independent mechanisms. Small bioactive molecules such as lipids and amino acids, produced by host cells or the gut microbiota, can also serve as intermediates in biochemical reactions or activate specific receptors and transcription factors, thereby impairing insulin sensitivity, disrupting glucose metabolism, compromising immune homeostasis, and ultimately contributing to renal dysfunction.

Deciphering the signaling network centered on metabolites requires research strategies that go beyond traditional metabolic profiling. Advances in multidimensional metabolomics technologies have enhanced our ability to precisely characterize metabolic changes in both spatial and temporal dimensions. Spatial metabolomics, such as matrix-assisted laser desorption/ionization mass spectrometry imaging (MALDI-MSI), enables the *in situ* localization of metabolites in specific kidney regions including the glomeruli, tubules, and interstitium, thereby revealing the heterogeneity of metabolic abnormalities within the kidney (11, 12). Metabolic flux analysis (MFA) using stable isotope tracers overcomes the limitation of static metabolite concentration measurements. It allows tracing of the dynamic activity of metabolic pathways and distinguishes alterations in metabolite synthesis from those in utilization (13, 14). Furthermore, the multi-omics research approach, which combines metabolomics with genomics, transcriptomics, proteomics, metagenomics, and other omics technologies, can systematically analyze the abnormal regulation of metabolic networks in DKD (15, 16). The application of these technologies has shifted the field from descriptive studies of metabolic changes toward in-depth mechanistic elucidation of metabolic networks.

We performed a comprehensive search in databases including PubMed and Web of Science. The aim was to identify articles related to diabetic kidney disease. The search period extended from the earliest available records to August 2026. The following keywords were used: “diabetic nephropathy”, “diabetic kidney disease”, “metabolomics”, “metabolites”, “biomarkers”, and “mechanisms”. Original research articles and relevant reviews published in English were included. Building on the selected literature, this review takes metabolites as the core perspective and integrates existing research findings. We propose that small molecule metabolites are not only passive biomarkers, but also active driving factors in the pathogenesis of DKD, as well as therapeutic targets for intervention. This review elaborates on the core mechanisms by which metabolites mediate kidney injury, with a focus on the AGEs–RAGE signaling axis, lipotoxic metabolites, amino acid metabolic reprogramming, and EV-mediated cell communication. We propose that these pathways collectively form an interconnected metabolic signaling network. We also evaluate the clinical application value of metabolite biomarkers, which lies in three aspects: early disease detection, prediction of disease progression, and investigation of pathogenic mechanisms. Furthermore, we discuss novel therapeutic strategies targeting specific metabolic nodes. We propose a hypothetical roadmap toward metabolic precision medicine, in which patients are stratified according to their distinct metabolic phenotypes to guide individualized treatment. This framework aims to provide a new theoretical basis for overcoming the current bottlenecks in the diagnosis and treatment of DKD.

Several recent reviews have discussed metabolomics and specific metabolic pathways in DKD. This review differs in two key ways. First, it does not treat metabolic pathways as parallel or isolated entities. It proposes a self-amplifying metabolic signaling network. It systematically defines the network nodes, including AGEs, ceramides, and BCAAs. It also defines the network connections, including EV-mediated transfer and mTOR complex 1 (mTORC1) integration. These nodes and connections show the organ-wide spread of local metabolic insults. Second, it goes beyond traditional descriptive lists of biomarkers. It proposes an actionable, hypothesis-generating framework for metabolic precision medicine. This framework stratifies patients into specific metabolic subtypes. It maps these subtypes directly to mechanism-based therapeutic nodes. This approach provides a new paradigm for clinical translation.

## 2 Metabolites are hallmarks of DKD

The pathological changes in DKD result from a complex array of interacting metabolic insults, with contributing factors extending well beyond hyperglycemia. Here, we introduce four interrelated core mechanisms that together form a self-sustaining and intrarenally propagating metabolic signaling network. First, extracellular metabolites, such as AGEs, bind to plasma membrane receptors, thereby initiating the injury process. Second, the intracellular accumulation of lipotoxic metabolites amplifies cellular stress signals. Third, aberrant alterations in amino acid metabolism remodel core pathways governing cell survival and growth. Fourth, these intracellular metabolic disturbances are packaged into EVs, through which pathogenic metabolites and signals can be transmitted to neighboring as well as distant cells, thereby coordinating the renal response to local metabolic stress across the entire kidney.

### 2.1 Activation of the AGEs–RAGE signaling pathway

Advanced glycation end products are irreversible derivatives formed by non-enzymatic reactions among proteins, lipids, and nucleic acids under diabetic hyperglycemic conditions. Their abnormal accumulation in the kidney represents a core pathogenic mechanism of DKD (17–19). More importantly, the receptor-dependent pathway involves the binding of extracellular AGEs to their primary receptor RAGE, leading to the activation of multiple downstream signaling pathways (20, 21). First, the AGEs–RAGE interaction triggers phosphorylation and translocation of protein kinase C (PKC, particularly the PKC-δ isoform). Activation of PKC-δ promotes the transcriptional upregulation of Nox4, which is highly expressed in the kidney, thereby leading to excessive ROS production and enhanced oxidative stress (22, 23). Second, the AGEs–RAGE interaction recruits intracellular adaptors that initiate a kinase cascade, leading to the sequential phosphorylation and activation of p38 MAPK. Concurrently, this signaling axis induces the phosphorylation-dependent degradation of IκB, thereby releasing NF-κB for nuclear translocation. These coordinated molecular events collectively drive the transcriptional upregulation of numerous pro-inflammatory factors, including TNF-α, IL-1β, IL-6, and MCP-1, resulting in sustained renal inflammatory responses and cellular apoptosis (24, 25). Third, AGEs–RAGE signaling engages in crosstalk with the TGF-β/Smad3 axis and stimulates the MEK-dependent phosphorylation of ERK1/2. These integrated signals converge on the promoter regions of fibrotic genes, significantly upregulating the expression of extracellular matrix components such as fibronectin, collagen I/IV, and α-SMA, thereby contributing to glomerular mesangial matrix expansion (26–28). Recent studies have further demonstrated that AGEs induce podocyte injury and apoptosis, suppressing the expression of key slit diaphragm proteins such as nephrin and podocin, which leads to podocyte depletion and disruption of the glomerular filtration barrier integrity, ultimately contributing to the development of proteinuria (29). Thus, the AGEs–RAGE axis constitutes a central pathway in the pathological advancement of DKD by integrating inflammatory, oxidative stress, and fibrotic signals.

### 2.2 Abnormal accumulation of lipotoxic metabolites

Lipid metabolism disorder in DKD extends beyond the traditional systemic level and involves-an abnormal buildup of lipids in the kidney. These lipid metabolites, such as free fatty acids (FFAs), ceramides, diacylglycerol (DAG), and cholesterol, drive renal injury through direct cytotoxic effects and act as signaling molecules (30–32). The core mechanism initiates with mitochondrial dysfunction: hyperglycemia and elevated circulating FFAs promote excessive uptake of FFAs by renal tubular cells, leading to impaired mitochondrial function and promoting esterification of unoxidized FFAs into toxic lipid species (33, 34). Ceramide accumulation constitutes a critical pathogenic event because it induces mitochondrial ROS production, promotes the opening of the mitochondrial permeability transition pore and the release of cytochrome c, directly activating the caspase cascade and triggering apoptosis. Moreover, it inhibits phosphorylation of Akt (protein kinase B), thereby impairing critical cell survival signaling pathways (35, 36). The accumulation of another key lipotoxic metabolite, DAG, acts as a potent activator of PKC. Activation of the DAG-PKC axis (particularly PKC-α and PKC-β) mediates damage through the following mechanisms: upregulation of TGF-β expression, promoting extracellular matrix synthesis and fibrosis; activation of NADPH oxidases (Nox4 and Nox1), further exacerbating renal oxidative stress; and induction of serine phosphorylation of insulin receptor substrate 1 (IRS-1), leading to reduced PI3 kinase activity and consequent local insulin resistance in the kidney (37–39). In addition, abnormal cholesterol accumulation in renal cells, especially podocytes and mesangial cells, alters the structure and function of lipid rafts in cell membranes, providing a platform for the assembly of inflammatory signaling complexes (40, 41). Cholesterol crystals or other lipid stress signals can promote the activation of NLRP3 and caspase-1, increasing the secretion of pro-inflammatory cytokines, and triggering intense local inflammatory responses (42–44) (**Figure 2**). Finally, peroxisome proliferator-activated receptors (PPARs), of which PPARα is classically considered the master regulator of fatty acid oxidation (FAO) and PPARγ primarily regulates adipogenesis and lipid storage, exhibit downregulated activity in DKD. This further exacerbates defects in FAO and lipid accumulation, creating a vicious cycle (45, 46). Therefore, renal lipotoxicity is a complex process driven by the synergistic effects of multiple lipid metabolites, collectively promoting DKD progression through pathways involving oxidative stress, apoptosis, inflammation, and fibrosis.

**Figure 2:**
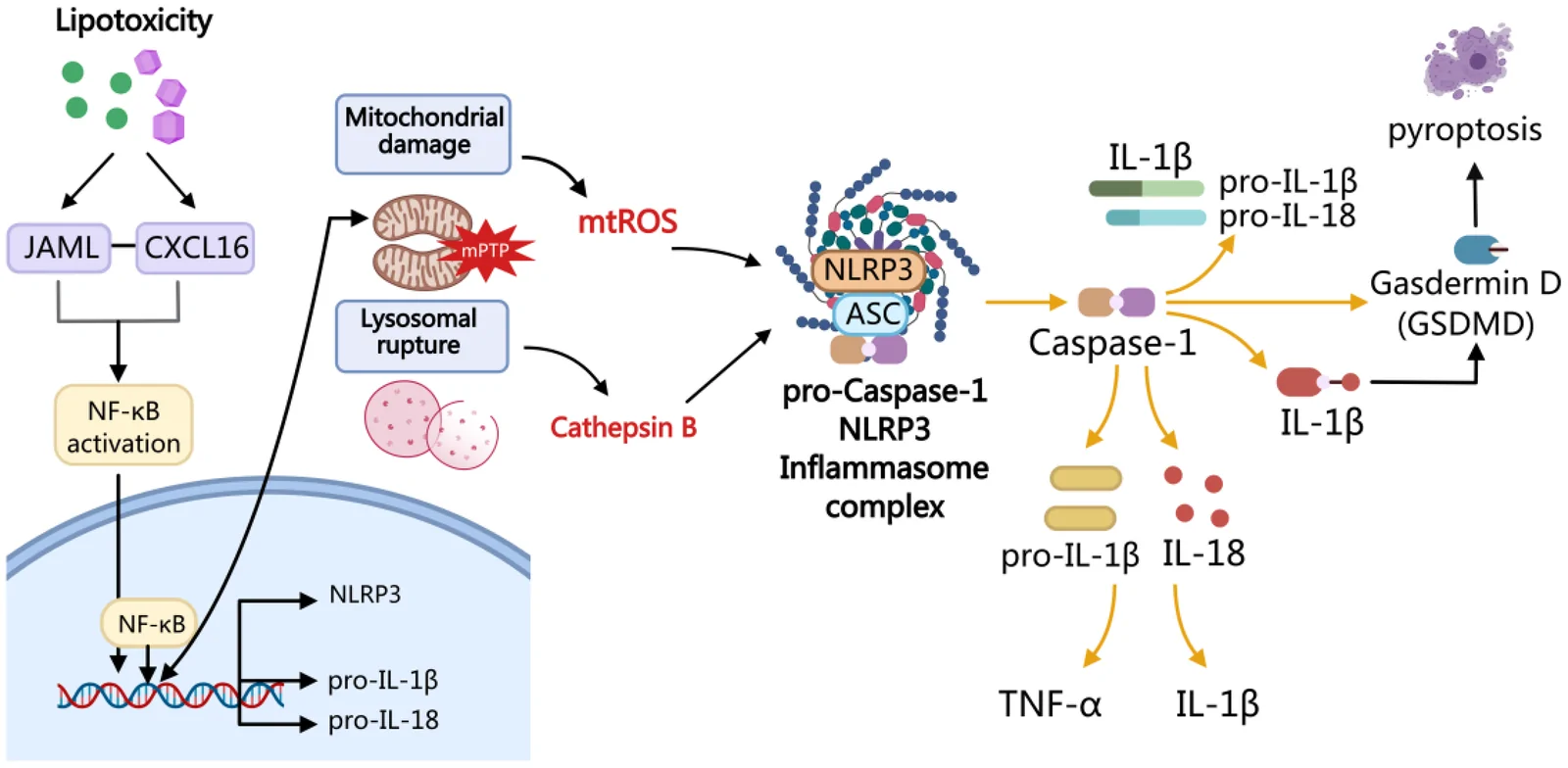
Lipid signaling promotes NLRP3 inflammasome activation and pyroptosis. Lipotoxic stress signaling induces JAML expression and functional activation in macrophages. Upregulated JAML activates the NLRP3 inflammasome and NF-κB pathway, leading to mitochondrial ROS release and lysosomal cathepsin B leakage. This triggers assembly of the NLRP3-ASC-caspase-1 complex, resulting in maturation and release of IL-1β/IL-18 and an inflammatory response. Activated caspase-1 not only promotes maturation of proinflammatory cytokines but also cleaves gasdermin D to form membrane pores, inducing pyroptosis. This image was created with MedPeer (http://www.medpeer.cn).

### 2.3 Reprogramming of amino acid metabolism

Dysregulation of the metabolic profile of amino acids has emerged as a novel molecular link connecting metabolic abnormalities in diabetes to renal complications (47, 48). Glutamate is a multifunctional molecule in the peripheral organs and is a dominant neurotransmitter. In mitochondria, glutamate is oxidatively deaminated by glutamate dehydrogenase (GDH) to α-ketoglutarate, which enters the TCA cycle to support energy production, whereas the plasma membrane cystine/glutamate antiporter xCT (SLC7A11) regulates cell proliferation and maintains redox homeostasis in renal cells by exchanging extracellular cystine for intracellular glutamate (49, 50) (**Figure 3**). Circulation and tissue levels of branched-chain amino acids (BCAAs) are elevated under insulin-resistant conditions, and the core pathogenic mechanism involves sustained activation of mTORC1 signaling (51, 52). A recent paper mentions that it is the impaired catabolism of BCAAs which may drive kidney pathology, with MCP-1 mediating over 50% of this association (53). Leucine, as a key activator of mTORC1, recruits and activates mTORC1 at the lysosomal membrane via a Rag GTPase-dependent mechanism (54). Hyperactivation of mTORC1 causes multiple detrimental effects. First, it potently suppresses cellular autophagy, impairing the clearance of damaged mitochondria and misfolded proteins, thereby exacerbating intracellular stress and apoptosis sensitivity (55). Second, it promotes protein synthesis and cellular hypertrophy, directly contributing to glomerular hypertrophy (56, 57). Third, sustained mTORC1 activation promotes S6K1 activity. S6K1 phosphorylates IRS-1 on serine residues, which impairs IRS-1 tyrosine phosphorylation and its association with the insulin receptor, reduces PI3K/Akt signaling, and thereby contributes to renal insulin resistance (58, 59). In addition, intermediates generated by BCAAs catabolism (such as acylcarnitines) accumulate within mitochondria, which can directly impair mitochondrial function and increase ROS production (60–62). On the other hand, activation of the kynurenine (Kyn) pathway of tryptophan metabolism represents another important mechanism (63–65). In the inflammatory milieu of DKD, cytokines such as interferon-gamma (IFN-γ) induce the expression of indoleamine 2,3-dioxygenase (IDO1), which shifts tryptophan metabolism toward excessive production of Kyn (66, 67). Activation of the aryl hydrocarbon receptor (AhR) by the endogenous ligands Kyn and kynurenic acid directly transcriptionally upregulates TGF-β1, thereby driving renal fibrosis (68–70). More importantly, the intermediate metabolite 3-hydroxykynurenine in this pathway is a potent oxidant that can directly induce oxidative stress and apoptosis, causing tubular damage independent of receptor-mediated signaling (71–73). Clinical studies have further confirmed that plasma levels of BCAAs and Kyn are significantly associated with the onset and progression of DKD (74, 75).

**Figure 3:**
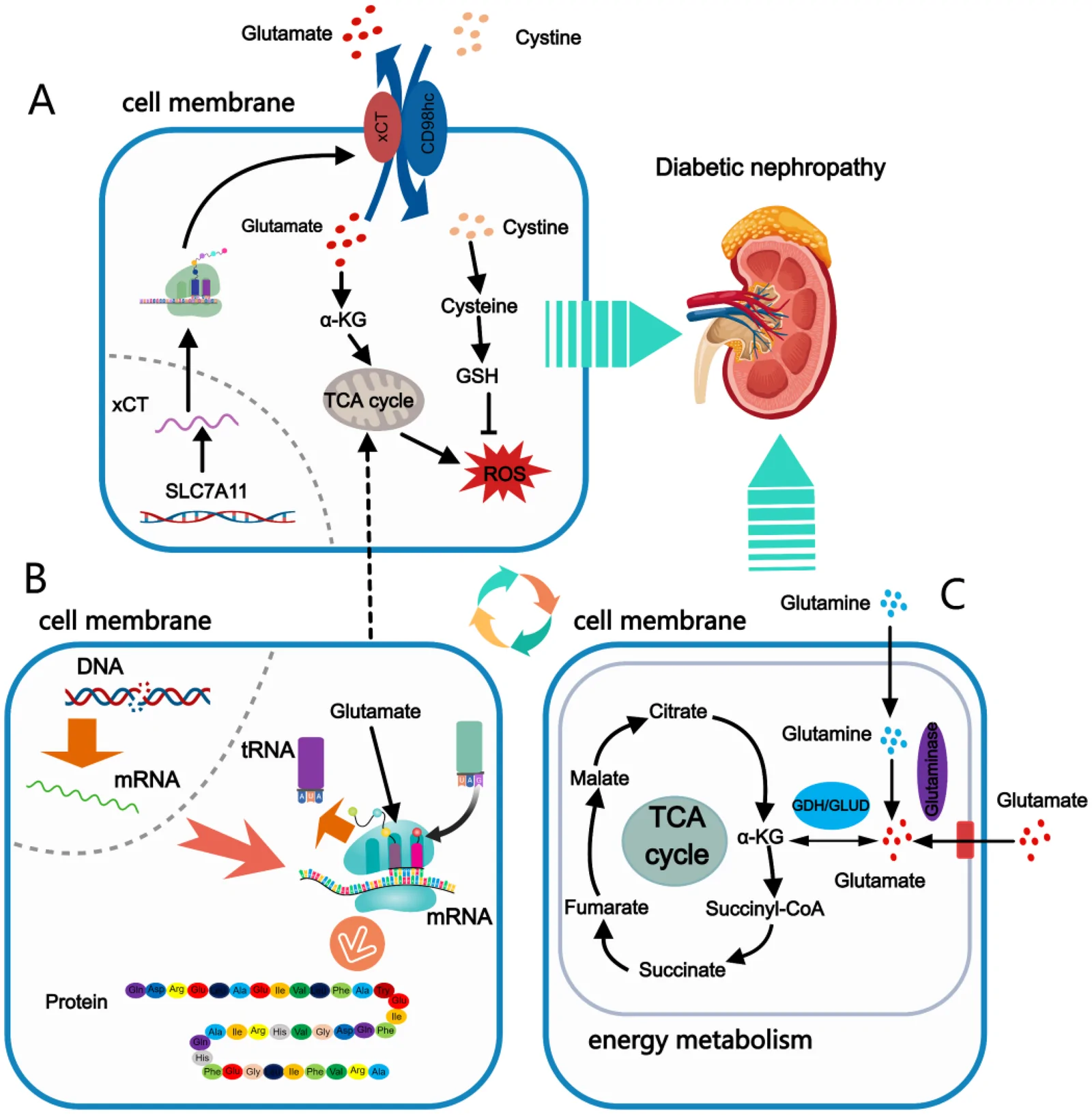
Physiological mechanisms of glutamate in DKD. **(A)** The cystine/glutamate antiporter xCT (SLC7A11) regulates cell proliferation by mediating the exchange of extracellular cystine for intracellular glutamate, thereby maintaining cellular redox homeostasis and supporting energy metabolism. This transport system is crucial for renal antioxidant defense. **(B)** Glutamate is not only a substrate for protein synthesis but also a key metabolite that supports fundamental cellular functions. **(C)** In mitochondria, glutamate is oxidatively deaminated by GDH to yield α-ketoglutarate, which enters the TCA cycle and is oxidized to generate reducing equivalents for ATP production. This image was created with MedPeer (www.medpeer.cn). .

BCAAs activate mTORC1, and tryptophan metabolites induce AhR signaling. These findings establish the central regulatory roles of these amino acid pathways in cell growth, autophagy, and inflammatory responses. Recent studies indicate that microRNAs (miRNAs) associated with metabolic stress responses are selectively enriched in EVs derived from stressed renal cells. For instance, miR-21, which is mechanistically linked to both mTORC1 and TGF-β signaling, can be packaged into EVs (76, 77). These findings raise the possibility that amino acid metabolic reprogramming may influence EV cargo and thereby affect the broader renal microenvironment. However, whether this represents a direct causal cascade from BCAA/mTORC1 or AhR activation to specific EV miRNA sorting requires further experimental validation. Thus, amino acid metabolic reprogramming is deeply involved in the pathophysiology of DKD, influencing cell proliferation, insulin signaling, autophagy, oxidative stress, and fibrosis, while also affecting EV-mediated cell communication.

### 2.4 EVs: dynamic carriers of the metabolic signaling network

In the preceding sections, we have detailed how different classes of metabolites (e.g., AGEs, lipids, and amino acids) drive intracellular stress pathways. Consequently, kidney injury in DKD is not confined to the initially stressed cells. It spreads to adjacent nephrons and also affects distant nephrons. Recent studies have revealed that EVs, including exosomes, microvesicles, and apoptotic vesicles, function as specific carriers of metabolites and establish a complex “metabolic communication network” among various renal cell types, such as renal tubular cells, podocytes, mesangial cells, and immune cells, thereby amplifying injury signals and coordinating the progression of DKD (78–81). Under stimulation by hyperglycemia, AGEs, and inflammatory factors, resident renal cells and infiltrating immune cells secrete increased amounts of EVs. These EVs not only carry proteins and nucleic acids (e.g., miRNAs and lncRNAs), but also selectively encapsulate a variety of biologically active metabolites, such as TCA cycle intermediates and prostaglandins (82–84). First, EVs mediate “remote toxicity” through metabolite delivery. Indoxyl sulfate and p-cresyl sulfate, which are uremic toxins produced by the gut microbiota, stimulate intestinal epithelial cells and circulating immune cells to generate EVs enriched with specific miRNAs (e.g., miR-92a) and inflammatory factors. These EVs subsequently induce inflammation, apoptosis, cellular senescence, proliferation, calcification, and neointimal hyperplasia (85–88). Second, EVs create a unique metabolic microenvironment. In the diabetic context, these EVs exacerbate endothelial dysfunction and vascular inflammation, promote the secretion of pro-inflammatory cytokines, potentially contributing to the development of glomerular inflammation and proteinuria *in vivo* (89, 90). More importantly, miRNAs carried within EVs (such as the pro-fibrotic miR-21) can simultaneously suppress multiple protective target genes and interact with signaling axes including TGF-β/NF-κB pathways, forming an integrated metabolic network that promotes cytoskeletal remodeling, extracellular matrix deposition, fibrosis, and inflammation (91–93). In addition, emerging evidence on inter-organelle communication indicates that mitochondria-derived vesicles or even intact extracellular mitochondria can be released into circulation. The mtDNA they carry mediates systemic and renal inflammatory responses (94, 95).

Thus, EVs receive signals from different metabolic stress pathways, including glycation, lipotoxicity, and amino acid imbalance, and propagate these signals throughout the kidney. This metabolic signal transmission mediated by extracellular vesicles transforms the originally localized cellular stress response into a coordinated, organ-wide pathological process. Ultimately, it drives the kidney from initial metabolic damage to irreversible structural damage. Reducing the secretion of extracellular vesicles by inhibiting relevant metabolic enzymes, or engineering vesicles to deliver protective metabolites, offers a novel precise intervention strategy for DKD (96, 97).

In summary, the four core mechanisms discussed above do not operate independently. Together, they constitute an integrated, self-amplifying network that drives the progression of DKD. This network perspective has changed our understanding of DKD. In the past, we viewed it as a series of parallel independent mechanisms, but now we see it as a systemic metabolic disorder. We need to define the structural components of this network model. This definition is essential to establish the model rigorously. The “nodes” are key bioactive metabolites or their intracellular sensors. They initiate or sustain pathological signals. Proposed nodes include AGEs, ceramides, DAG, BCAAs, and trimethylamine N-oxide (TMAO). The “connections” are biochemical or biophysical interactions. They allow these nodes to propagate signals. Examples include metabolite-mediated activation of the metabolic pathway, acetyl-CoA-driven epigenetic reprogramming, and intercellular transfer of toxic cargo via EVs. Several proposed nodes and connections have support from human studies. For example, kidneys from patients with DKD show conserved dysregulation of glycerolipid metabolism and MAPK signaling. Spatial multi-omics analysis revealed that 179 and 234 differentially expressed genes were enriched in the MAPK pathway. This study provides direct evidence for spatial crosstalk between metabolic reprogramming and inflammatory signaling in human kidney tissue (98). ATP-citrate lyase (ACLY) converts citrate into acetyl-CoA and supplies substrate for histone acetylation. Kidney biopsy tissues from patients with chronic kidney disease show increased histone H3K27ac acetylation and elevated tissue citrate. ACLY is significantly upregulated in renal tubular epithelial cells. Its expression is negatively correlated with eGFR in patients (99). Thirteen EV miRNAs and 17 target mRNAs, including RRM2, NT5E, and UGDH, were commonly differentially expressed in plasma and urine EVs from human patients with DKD. The target pathways of these EV miRNAs are involved in metabolic networks. Urinary succinate and adenosine concentrations are associated with DKD progression (100). Urinary EVs (uEVs) from patients with T1DM show a more negative zeta potential and a higher particle concentration. ToF-SIMS showed measurable differences in lipid ions such as diacylglycerol, sphingolipids, and specific fatty acids. Amino acid-related ions also showed slight changes. This study suggests that uEVs can reflect pathological processes in patients with T1DM (101). These human data provide a translational foundation for the proposed network interactions. In this disorder, various metabolic abnormalities reinforce each other. This also means that effective treatment may require targeting network hubs such as metabolites or EVs, or simultaneously targeting multiple nodes, rather than just focusing on a single isolated pathway.

## 3 Advanced technology platform for metabolites

To analyze the complex metabolic signaling network in DKD, we need advanced analytical methods beyond traditional metabolite profiling. We need a multidimensional metabolic toolkit that integrates multiple complementary technologies. This toolkit can clearly depict the nodes, connectivity relationships, metabolic flow, and spatial and dynamic changes of the network. Below, we will provide a detailed introduction to the core technologies of multidimensional metabolomics and how their integrated applications can enhance our ability to analyze DKD metabolic mechanisms (**Figure 4**).

**Figure 4:**
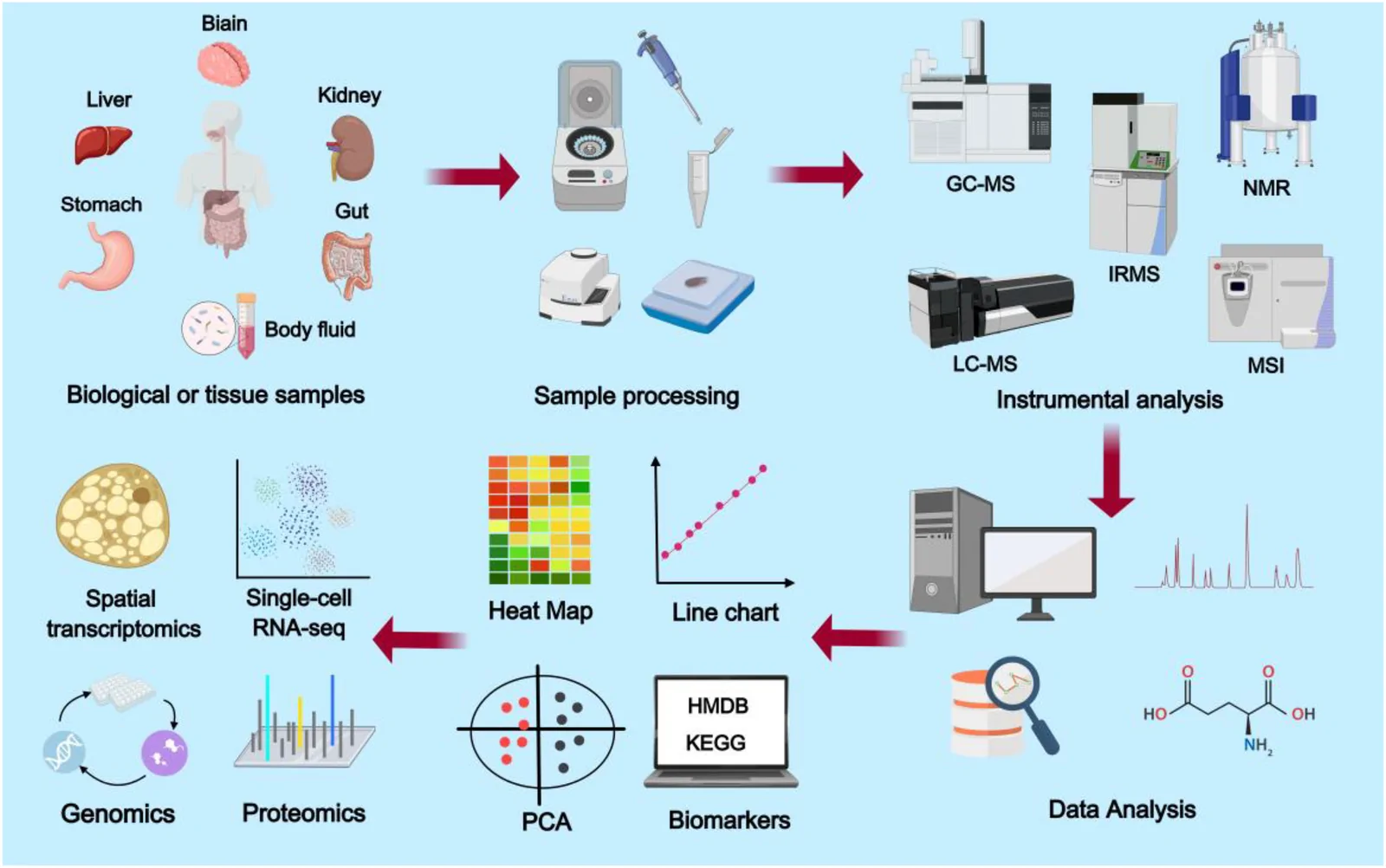
Integrated multi-technology analysis process for metabolites. Biological fluids such as blood, urine, and cerebrospinal fluid are collected; liver, kidney, and other tissues (including intestinal flora) are isolated and extracted; and processed samples are analyzed using advanced technology platforms such as LC-MS, NMR, MSI, IRMS, and GC-MS to generate important information and characteristics of small-molecule metabolites. Finally, the collected data are analyzed using multi-omics integration to identify disease-specific biomarkers and pathogenesis. This image was created with MedPeer (www.medpeer.cn). .

### 3.1 Compositional profiling based on untargeted and targeted metabolomics

The first step in decoding a metabolic network is to define its baseline composition. We need to identify which metabolites are altered in DKD and quantify their concentrations. Liquid chromatography–mass spectrometry (LC-MS) and gas chromatography–mass spectrometry (GC-MS) have become the main tools for such studies because of their high sensitivity, broad coverage, and ability to handle complex biological samples such as blood, urine, and tissue extracts (102–104). Nuclear magnetic resonance (NMR) allows absolute quantification of metabolites using a single internal standard and serves as a key technique for elucidating the stereochemical structures of unknown compounds through chemical shifts, coupling constants, and multi-dimensional spectral analysis. When combined with stable isotope labeling, NMR can also track metabolic transformations and map pathway topologies (105, 106). In summary, MS and NMR-based analyses have generated a broad map of metabolite changes in DKD, systematically documenting alterations in various metabolite classes, including amino acids, lipids, and organic acids, in biofluids and tissues. However, these compositional maps are static snapshots that can only reveal correlations. They cannot distinguish whether such accumulation results from increased production, decreased consumption, or altered transport. This limitation requires dynamic and spatial approaches to address.

### 3.2 Dynamic tracking via metabolic flux analysis

To move beyond static concentration measurements and understand the flow of metabolites through pathways, metabolic flux analysis (MFA) using stable isotope tracers (such as ¹³C and ²H) has become indispensable (107, 108). The breakthroughs offered by MFA are essential for understanding the pathogenesis of DKD (109, 110). For example, conventional metabolomics might reveal accumulation of TCA cycle intermediates in the diabetic kidney, but only MFA can determine whether this results from increased anaplerotic influx (e.g., from glutamine) or decreased oxidative efflux due to mitochondrial dysfunction (111, 112). In DKD research, MFA has been less widely used than other omics technologies, but it has already begun to yield fundamental insights. Studies using ¹³C-labeled glucose or glutamine in cell and rodent models have identified sex-specific shifts in substrate utilization. For instance, under high-glucose conditions, male-derived proximal tubular epithelial cells tend to divert glucose and glutamine into the TCA cycle, a sex-specific metabolic feature associated with DKD progression (113). An emerging technique that combines stable isotope infusion with MSI can even visualize regional differences in metabolic activity within intact tissues, thereby adding a spatial dimension to flux analysis (114). Despite its power, MFA remains underutilized in DKD research, partly because of the complexity of *in vivo* tracer studies, the need for specialized computational modeling, and the difficulty of translating findings to human cohorts. Nevertheless, integrating MFA with conventional metabolomics is critical for moving from descriptive biomarker discovery to mechanistic pathway validation.

### 3.3 Spatial localization by metabolite imaging

The kidney is a highly heterogeneous organ, with distinct metabolic profiles and functions across the glomeruli, tubules, and interstitium. Conventional metabolomics requires homogenization of whole tissues, which averages this spatial heterogeneity and masks the cellular origins of metabolic changes. MSI overcomes this limitation by enabling *in situ* identification and spatial localization of metabolites in tissue sections at micrometer resolution (115). This label-free, untargeted technique allows simultaneous characterization of known compounds, discovery of novel metabolites, and generation of spatial maps of metabolite distribution (116, 117). MSI has revealed distinct spatial patterns of metabolic injury in DKD. Tanaka et al. used MALDI-MSI to analyze kidneys from 22-week-old BTBR ob/ob mice and found that citrate and oxidized glutathione accumulated specifically in the glomerular region of the renal cortex, pinpointing glomerular mitochondria as a center of oxidative stress in DKD (118). Zhang et al. employed MSI to map metabolite distributions in DKD kidneys, revealing spatial distribution patterns of AMP, eicosapentaenoic acid, and various phospholipids across different renal regions (119). Furthermore, when combined with stable isotope infusion under isotopic steady-state conditions, MSI can even visualize and quantify regional differences in metabolic activity, allowing direct comparison of pathway utilization across distinct tissue regions (114). By adding the “where” to the “what” and “how much”, spatial metabolomics bridges metabolic biochemistry and histopathology. It provides a powerful tool for identifying cell type-specific drivers of DKD and advancing precision therapeutic strategies.

### 3.4 Integrative analysis through multi-omics and data fusion

Metabolites do not act in isolation. They are downstream products of genomic, transcriptomic, and proteomic alterations and are also profoundly influenced by environmental factors such as diet and the gut microbiota. Therefore, to fully understand the metabolic signaling network in DKD, we need to integrate metabolomics data with other omics datasets—a multi-omics approach (11, 120). Integrating metabolomics with genomics and transcriptomics can reveal how genetic variation and changes in gene expression drive metabolic alterations. For example, combined transcriptomic and metabolomic analysis identified UPP1 as a key regulator of uridine metabolism in DKD and confirmed that reduced uridine levels are central to disease progression (121). Integrating metabolomics with metagenomics is particularly important for understanding the impact of the gut microbiota on host metabolism. For instance, the choline-utilization C/D genes (cutC/D) encode choline trimethylamine (TMA) lyase, a key enzyme by which the gut microbiota converts dietary choline and carnitine into TMA. TMA is then absorbed into the portal circulation and transported to the liver, where it is oxidized to TMAO by host flavin-containing monooxygenase 3 (FMO3) (122, 123). Integrated analysis of the microbiome and metabolome in cohorts of patients with DKD revealed that these patients had reduced microbial diversity and abundance, and that altered bacterial taxa were associated with increased TMAO production. In a DKD mouse model, TMAO drove kidney injury by suppressing PRSS3 expression, and overexpression of PRSS3 significantly alleviated kidney injury (124). Moreover, advanced computational methods such as network analysis and machine learning are increasingly being used to fuse these different types of data (125). By constructing metabolite-protein interaction networks, researchers can identify functional targets directly modulated by metabolites, such as allosteric sites on metabolic enzymes or metabolite-sensing receptors (126, 127). As we move toward precision medicine, such integrative analysis is essential for building a holistic view of DKD pathogenesis—one that captures interactions among host genetics, the gut microbiota, diet, and the metabolic environment, and that can uncover hidden causal relationships within complex multi-omics data.

In summary, untargeted metabolomics generates hypotheses by revealing global metabolic changes. Targeted metabolomics validates and quantifies these candidate metabolites. MFA uncovers underlying pathway dynamics and distinguishes cause from effect. MSI localizes metabolic changes to specific cell types and tissue regions, revealing anatomical heterogeneity. Multi-omics integration links metabolites to upstream genetic, transcriptional, and microbial regulators. Each technique addresses a different dimension of the metabolic signaling network, and their combined application enables a shift from descriptive classification toward mechanistic, spatially resolved, and systems-level understanding.

## 4 Metabolites as functional biomarkers

Clinical management of DKD still relies on two traditional biomarkers, albuminuria and eGFR, which primarily detect established injury rather than early metabolic disturbances, thereby limiting opportunities for timely intervention (3). Some patients with normal albuminuria may experience progressive decline in kidney function, while others with elevated albuminuria exhibit divergent disease trajectories (128). Moreover, discordance between albuminuria and eGFR can occur in advanced disease, as structural glomerulosclerosis and tubulointerstitial fibrosis progress independently of permeability changes, meaning that a single marker cannot fully capture the complexity of DKD progression (129). This heterogeneity indicates that DKD is not a single disease but a spectrum of disorders driven by distinct pathophysiological processes, many of which arise from metabolic dysregulation. Metabolites offer a way to address this dilemma. As end products of metabolic pathways and active participants in metabolic signaling networks, metabolites can reflect both the state and the drivers of disease (130, 131). However, not all metabolite biomarkers have the same value; their clinical significance depends on their ability to detect disease before traditional indices become abnormal, predict rapid progression in patients, reveal mechanisms, and guide therapy.

From a functional perspective, we synthesize the literature on metabolite biomarkers in DKD (see **Tables 1**, **2**) and classify them into early detection markers, progression prediction markers, and pathogenic driver markers. This classification is a conceptual framework rather than a rigid taxonomy. The categories are non-mutually exclusive and context-dependent, and the same metabolite may serve different functions across disease stages, populations, and clinical settings. Early detection biomarkers are detectable before conventional indicators become abnormal and predict subsequent disease onset. Progression-predictive biomarkers are associated with longitudinal outcomes such as disease progression, complications, and death. Pathogenic driver biomarkers actively participate in disease mechanisms and may serve as therapeutic targets. This framework is intended to help clinicians and researchers select biomarkers according to specific clinical needs and to illustrate how multidimensional metabolomics can facilitate such functional classification and interpretation.

**Table 1:** Small molecule metabolites and related metabolic alterations in DKD clinical studies.

Population and size	Evidence level	Sample	Applied method	Research aim	Metabolites and trends of change	Metabolic pathway	Main outcomes	Effect size (95% CI)	AUC values	Sensitivity and specificity	Adjustment for confounders	Int/Ext validation	Refs
T1D with DKD (n=2670)	Prospective, observational study	Urine	NMR	Phenotype	L-valine↑, l-isoleucine↑, pseudouridine↑, citrate↓	Amino acid metabolism	Albuminuria	L-valine (1.38 [1.22, 1.56]), isoleucine (1.33 [1.18, 1.50]), pseudouridine (1.25 [1.11, 1.42])	NR	NR	Baseline albuminuria and CKD stage	NR	(132)
T2D with DKD (n=144)	Case–control study	Urine	LC-MS	Diagnosis	Tetranor-PGEM↑, maresin-1↓, leukotriene B4-ethanolamide↓	Lipid metabolism	Clinical parameters	NR	Tetranor-PGEM: 0.791/0.657	Tetranor-PGEM: 66.7/87.9%, 83.3/67.9%, 76.2/71.5% at 20.7/21.5/30.4 ng/mgCr	OPLS-DA included age, sex, eGFR, HbA1c, μAlb	NR	(158)
Chronic renal insufficiency (n=904)	Prospective cohort study	Urine, Kidney	LC-MS/MS MALDI-MSI	Diagnosis	Adenine↑	Purine metabolism	Urine adenine/Creatinine ratio	NR	NR	NR	NR	NR	(144)
Healthy, DKD (n=1513)	Prospective multicenter cohort	Serum	LC-MS/MS	Diagnosis	Glycerol-3-galactoside↑	Galactose metabolism, glycerolipid metabolism	Serum creat inine	NR	NR	HC vs DKD-A: 63.0/87.9% (Int), 88.5/100% (Ext-CS)	NR	Int test n=174; Ext test n=237 (3 cohorts)	(159)
T1D and T2D (n=462)	Prospective cohort	Plasma	UPLC-MS GC-MS	Phenotype	Histidyl-phenylalanine↑, leucylphenylalanine↑, tryptophylasparagine↑	Amino acid metabolism	eGFR	NR	NR	NR	Multivariable logistic regression adjusted for age, gender, BMI, etc.	NR	(160)
Healthy, T2D and DKD (n=90)	Case–control study	Serum	LC-MS/MS	Diagnosis	N-acetylaspartic acid↑, l-valine↑, l-isoleucine↑, l-asparagine↑, betaine↑, l-methionine↑	Amino acid metabolism	eGFR	L-valine HR 2.583 (1.006–6.629); isoleucine HR 1.670 (1.206–2.312) per one-point increment	Health vs T2DM:L-valine 0.943, Betaine 0.863	NR	Multivariable Cox: age, sex, BP, diabetes duration, etc.	NR	(161)
T2D and DKD (n=68)	Cross-sectional study	Urine	UPLC-MS	Diagnosis	Dihydrouracil↓, ureidopropionic acid↓, pantothenic acid↓, adenosine 3′,5′-diphosphate↓	Pantothenate and CoA biosynthesis	N/A	NR	NR	NR	Baseline matched for age, diabetes duration, BMI, etc.	NR	(162)
Healthy, T2D and DKD (n=766)	Cross-sectional study	Urine	LDI-MS	Diagnosis	5-Methylfuran-2-carboxylic acid↓, l- threonine↓, malonic acid↓, l-serine↓, hypotaurine↓, etc.	Aminoacyl-tRNA biosynthesis, valine, leucine, and isoleucine biosynthesis, and pantothenate and CoA Biosynthesis	UACR, eGFR	NR	3-step validation: 0.978/0.879/0.929	Layer 1: Sens 98.28%, Spec 82.35%	No multivariable adjustment reported	Ext test n=265	(163)
T2D (n=39)	Cross-sectional study	Urine	UHPLC-MS	Treatment	Hippuric acid↓, l-phenylalanine↑, l-tryptophan↓, deoxycholic acid↑, 3-hydroxyhippuric acid↓, indole-3-carboxylic acid↓	Glycine metabolism, tryptophan metabolism, valine, leucine and isoleucine degradation, purine metabolism	eGFR	NR	NR	NR	No multivariable adjustment reported	NR	(164)
T2D (n=5144)	Prospective observational	Plasma	LC-MS/MS	Phenotype	Triacylglycerols↑, diacylglycerols↑, ceramides↑, deoxysphingolipid↑	Lipid metabolism	eGFR	NR	NR	NR	Age, sex, ethnicity, BMI, diabetes duration, etc.	3 independent cohorts (DC, DN, SMART2D)	(135)
T2D (n=3061)	Prospective cohort study	Urine	LC-MS	Phenotype	Citrate↑, fumarate↑, and malate↑	TCA cycle	eGFR	Fumarate 1.82 (1.17–2.82); malate 1.49 (1.05–2.11); citrate 0.83 (0.72–0.96)	NR	NR	Multivariable Cox: Age, sex, ethnicity, smoking, etc.	Ext test n=1235	(133)
DKD (n=20)	Cross-sectional case-control study with animal validation	Serum	LC-MS/MS	Treatment	N-acetyl-l-aspartic acid↑, deoxyribose↑, oxoglutaric acid↑, l-phenylalanine↑, fumaric acid↑	Alanine, aspartate and glutamate metabolism, TCA cycle,phenylalanine metabolism	N/A	NR	NR	NR	NR	Animal model validation	(165)
CRIC (n=995)	Prospective cohort study	Urine	CE-MS	Prognosis	L-valine↑, l-tryptophan↑, 3-(4-methyl-3-pentenyl)thiophene↑, betaine↑	Valine-leucine-isoleucine degradation, tryptophan metabolism, lysine degradation	eGFR, HbA1c	3-(4-methyl-3-pentenyl)thiophene: HR 1.65 (1.23–2.20)	C-statistic 0.85 (0.85–0.86) for best model	NR	Multivariable Cox: Age, sex, race, smoking, etc.	Int replication on ESKD; targeted CE-MS validation	(142)
Healthy, T1D (n=50)	Cross-sectional study	Kidney	MALDI-MSI	Phenotype	Succinic acid↓, α-ketoglutaric acid↓, fumaric acid↓	TCA cycle	HbA1c, BMI, GBM	α-KG −5.48 (−10.2, −0.73); succinic acid −8.66 (−16.5, −0.80)	NR	NR	Baseline NEFA and body fat percentage for NEFA suppression	NR	(166)

**Table 2:** Small molecule metabolites and related metabolic alterations in DKD animal models.

Animal models	Sample	Applied method	Metabolites and trends of change	Metabolic pathway	Refs
db/db mice	Kidney, serum	LC-MS	Taurine↓, hypotaurine↓, l-tryptophan↓, tyrosine↓	Taurine and hypotaurine metabolism, tryptophan metabolism,tyrosine metabolism	(167)
db/db mice	Kidney, serum	LC-MS/MS	N-acetylcysteine↓, kynurenic acid↑, d-serine↑↓, 5-hydroxy-l-tryptophan↑ etc.	Tryptophan metabolism, glycine, serine and threonine metabolism	(168)
High-fat diet (HFD) with streptozotocin (STZ) rat model	Serum	LC-MS	L-kynurenine↑, l-glutamic acid↑, guanidineacetic acid↑, l-histidine↓, d-proline↓, 5-hydroxytryptophan↑, phenylpyruvic acid↑ etc.	Tryptophan metabolism, alanine, aspartate and glutamate metabolism, arginine biosynthesis, tyrosine metabolism, phenylalanine, tyrosine and tryptophan biosynthesis, cysteine and methionine metabolism	(169)
STZ induced mouse model	Kidney	UPLC-Q Exactive MS	Lysophosphatidylcholines↑, lysophosphatidylethanolamines↑, phosphatidylcholines↑, sphingomyelins↑, ceramides↑ etc.	Glycerophospholipid metabolism, sphingolipid metabolism	(170)
HFD with STZ rat model	Serum	LC-MS/MS	Phytosphingosine↓, sphingosine↓, glycerophosphocholine↑, phosphatidylethanolamine↓, etc.	Glycerophospholipid metabolism, sphingolipid metabolism	(171)
HFD with STZ rat model	Kidney	LC-MS/MS	Monoacylglycerol (MGA) ↓, tetradecanedioic acid↓, acylcarnitine↓	Lipid metabolism	(172)
HFD + unilateral ne- phrectomy + STZ rat model	Serum	GC–MS	L-monopalmitin↓, 1-monostearin↓	Lipid metabolism	(173)
HFD with STZ Rat Model	Urine, kidney, serum	LC-Q/TOF-MS	Indole-3-carboxylic acid↓, eicosadienoic acid↑ etc.	Phospholipid, arachidonic acid, and pyrimidine metabolisms	(174)
HFD with STZ rat model	Urine, Serum	UPLC-Q/TOF-MS	Cetoleic acid↓, oleoyl glycine↓, stearoylcarnitine↑, glycohyocholic acid↑, phenylalanine↓	Arachidonic acid metabolism, phenylalanine metabolism, sphingolipid metabolism	(175)
db/db mice	Serum	LC-MS	PGH3↑, ceramide(d18:1/12:0) ↑, xanthosine 5-triphosphate↓	Arachidonic acid metabolism, sphingolipid metabolism, purine metabolism	(176)
db/db mice	kidney	LC-MS	Methionine↑, α-ketoglutarate↑, s-adenosyl methionine↑, s- adenosylhomocysteine↓, nicotinamide↑	TCA cycle methionine cycle	(177)
db/db mice	Urine, kidney	NMR	L-tryptophan↓, alanine, N-acetylglutamate↓, l-glutamate↓, glutamine↓, phenylalanine↓ etc.	Energy metabolism, amino acid metabolism, nucleic acid metabolism, membrane lipid composition	(178)
db/db mice	Serum, kidney	LC-MS, AP-MALDI-MSI	3-dehydrosphinganine↓↑, retinyl ester↑, and 9-cis-retinoic acid↑	Retinol metabolism, sphingolipid metabolism	(179)
STZ induced mice model	Serum	LC-MS	Palmitic acid↑, arachidonic acid↓, pantothenic acid↑, sphingosine-1-phosphate↑ etc.	Arachidonic acid metabolism, pantothenate and CoA biosynthesis, sphingolipid metabolism	(180)
STZ induced rat model	Serum	UHPLC-MS/MS	Biotin↓, acar 19:1↓, uridine↓, menadione↑, Lysopa 18:0↑ etc.	Arginine biosynthesis, biosynthesis of amino acids, aminoacyl-tRNA biosynthesis	(121)
STZ induced rat model	Urine	UPLC-Q-TOF-MS/MS	Phenylglyoxylic acid↓, hippuric acid↓, indole-3-acetic acid↑, p-Cresol sulfate↑ etc.	Phenylalanine metabolism, tryptophan metabolism, taurine and hypotaurine metabolism, tyrosine metabolism	(181)
db/db mice	Serum	UPLC-MS/MS	Taurolithocholic acid↓	Bile acid metabolism	(182)
STZ induced mice model	kidney	UPLC-Q-TOF MS	LysoPC(15:0/0:0) ↑, lysoPC(16:1(9Z)/0:0) ↑, lysoPC(17:0/0:0)↑etc.	Glycerophospholipid metabolism	(183)
BTBR ob/ob mice	kidney	UPLC-IMS-QToF	Hyocholic acid↑, indole-3-propionic acid↓	Steroid metabolism, glycerophospholipid metabolism	(184)
STZ induced rat model, db/db mice	kidney	AFADESI-MSI	AMP↑, eicosenoic acid↓, eicosapentaenoic acid↑, phosphatidylserine (36:1) ↑, phosphatidylserine (36:4) ↑	TCA cycle, lipid metabolism, carnitine metabolism, choline metabolism, purine metabolism	(119)
BTBR ob/ob mice	kidney	IC-MS, MALDI-MSI	Fumarate↑, citrate↑, oxidized glutathione↑	TCA cycle	(118)

However, when interpreting circulating metabolite concentrations as biomarkers or pathogenic mediators, renal clearance must be carefully considered. As kidney function declines in DKD, circulating levels of various metabolites may increase partly due to reduced renal elimination, rather than solely reflecting increased systemic production. This potential reverse causality is particularly important when linking metabolite concentrations to DKD severity or progression. Therefore, evaluating the ratio of metabolites in both urine and plasma, or normalizing metabolite levels to eGFR, can help distinguish between altered generation and impaired clearance.

### 4.1 Early detection biomarkers

An ideal early biomarker should be able to detect the risk of DKD or early injury before traditional markers become abnormal, thereby enabling preventive intervention. Amino acid disturbances appear early in the disease course. In prospective cohort studies of patients with type 1 and type 2 diabetes, elevated baseline levels of urinary BCAAs preceded the onset of albuminuria, and these metabolites predicted the development of DKD years before clinical diagnosis (53, 132). Stefan Mutter et al. followed 2,670 patients with type 1 diabetes for 9.0 ± 5.0 years and found that urinary leucine, valine, and isoleucine were early markers of incident albuminuria (132). TCA cycle intermediates also show early changes. In a prospective study of 1,826 patients with type 2 diabetes, urinary levels of pyruvate, citrate, α-ketoglutarate, succinate, fumarate, and malate were associated with subsequent eGFR decline. Fumarate and citrate predicted risk of progression to advanced kidney disease, and their predictive ability was independent of clinical risk factors and other urinary metabolites (133). Notably, these metabolites reflect mitochondrial function, suggesting that mitochondrial dysfunction occurs before clinically detectable kidney injury. Thus, these early detection markers capture metabolic stress including mitochondrial dysfunction, amino acid imbalance, and nucleotide dysregulation that occurs before, and may even drive, structur-l injury.

### 4.2 Progression prediction biomarkers

Once DKD is diagnosed, patients exhibit highly variable clinical trajectories. Some patients maintain stable kidney function for years, while others progress rapidly to ESRD. Biomarkers that predict disease progression can guide the intensity of monitoring and treatment, as well as facilitate patient selection for clinical trials of novel therapeutics. Lipid metabolites have emerged as strong predictors of disease progression. Xu et al., in a cohort of 577 individuals, identified a panel of three lipids, LPE(16:0), PE(16:0/20:2), and TAG54:2-FA18:1, that distinguished different stages of DKD and was associated with eGFR decline (134). Ceramides, particularly Cer(d18:1/16:0) and Cer(d18:1/24:1), have been consistently associated with progression to ESRD across multiple cohort studies (135–137). Gut microbiota-derived metabolites also show remarkable predictive ability. TMAO has been associated with kidney outcomes in human cohorts. In a study of 440 patients with type 2 diabetes, higher TMAO concentrations were associated with increased risks of serum creatinine doubling, progression to ESRD, and death (138). This association persisted after adjustment for eGFR, suggesting that TMAO provides prognostic information beyond traditional markers. Another microbial metabolite, phenyl sulfate, is associated with progression of microalbuminuria (139–141). Amino acid signatures also predict disease progression. In 995 participants from the Chronic Renal Insufficiency Cohort (CRIC) study, urinary valine, isoleucine, and tryptophan were associated with eGFR decline and progression to ESRD (142). What distinguishes progression prediction markers from early detection markers is their association with the rate of disease change. These metabolites not only identify the presence of disease but also quantify its trajectory, providing information on disease activity.

### 4.3 Pathogenic driver biomarkers

While clinical metabolomic studies primarily provide associative evidence, findings from experimental models suggest that certain metabolites actively drive kidney injury. Here, we discuss metabolites that have been mechanistically linked to DKD pathogenesis in preclinical studies, which may directly guide therapeutic strategies. If a metabolite drives kidney injury, lowering its level (or blocking its action) should confer therapeutic benefit. In addition to serving as an early detection marker, adenine has been shown in mechanistic studies using animal models to directly stimulate the mTORC1 pathway in renal cells, increase soluble TNFR1 and KIM-1, induce cell hypertrophy, and promote matrix production (143). Sharma et al. used MSI to localize adenine to pathological lesions in human DKD biopsy tissues, providing direct evidence for its involvement in tissue injury (144). These findings suggest that adenine is not only a biomarker but may also be a therapeutic target; interventions that reduce adenine production or block its action may ameliorate DKD. Animal studies have shown that phenyl sulfate induces albuminuria and podocyte injury, reduces mitochondrial function, thickens the glomerular basement membrane, and promotes inflammation (145, 146). Its levels correlate with the progression of microalbuminuria. Ornithine and its downstream metabolites illustrate the connection between metabolic reprogramming and structural pathology (147–149). Accumulating clinical evidence has indicated that ornithine levels correlate with disease progression in DKD, underscoring its potential as a predictive biomarker (150, 151). Mechanistic studies in animal models have shown that when glycolysis is impaired in podocytes, the ornithine catabolic pathway is activated. The intermediates generated remodel the podocyte cytoskeleton through the Rheb-mTOR signaling axis, ultimately leading to foot process effacement. Specific inhibition of ornithine catabolism attenuates podocyte injury, validating this pathway may as a potential therapeutic target (152) (**Figure 5**). Here, metabolites serve both as markers of pathway activation and as guides for intervention. TMAO is associated with disease progression in human cohorts. In preclinical models, TMAO has been reported to promote renal fibrosis, activate inflammatory pathways, and impair mitochondrial function. These findings suggest that TMAO may also act as a pathogenic mediator. However, whether these mechanisms are directly causal in patients remains to be established (153–155). In summary, one possible implication of these findings is that once a metabolite is confirmed as a pathogenic driver, its metabolic pathway may represent a druggable node, and its concentration may serve as a pharmacodynamic biomarker for monitoring treatment response.

**Figure 5:**
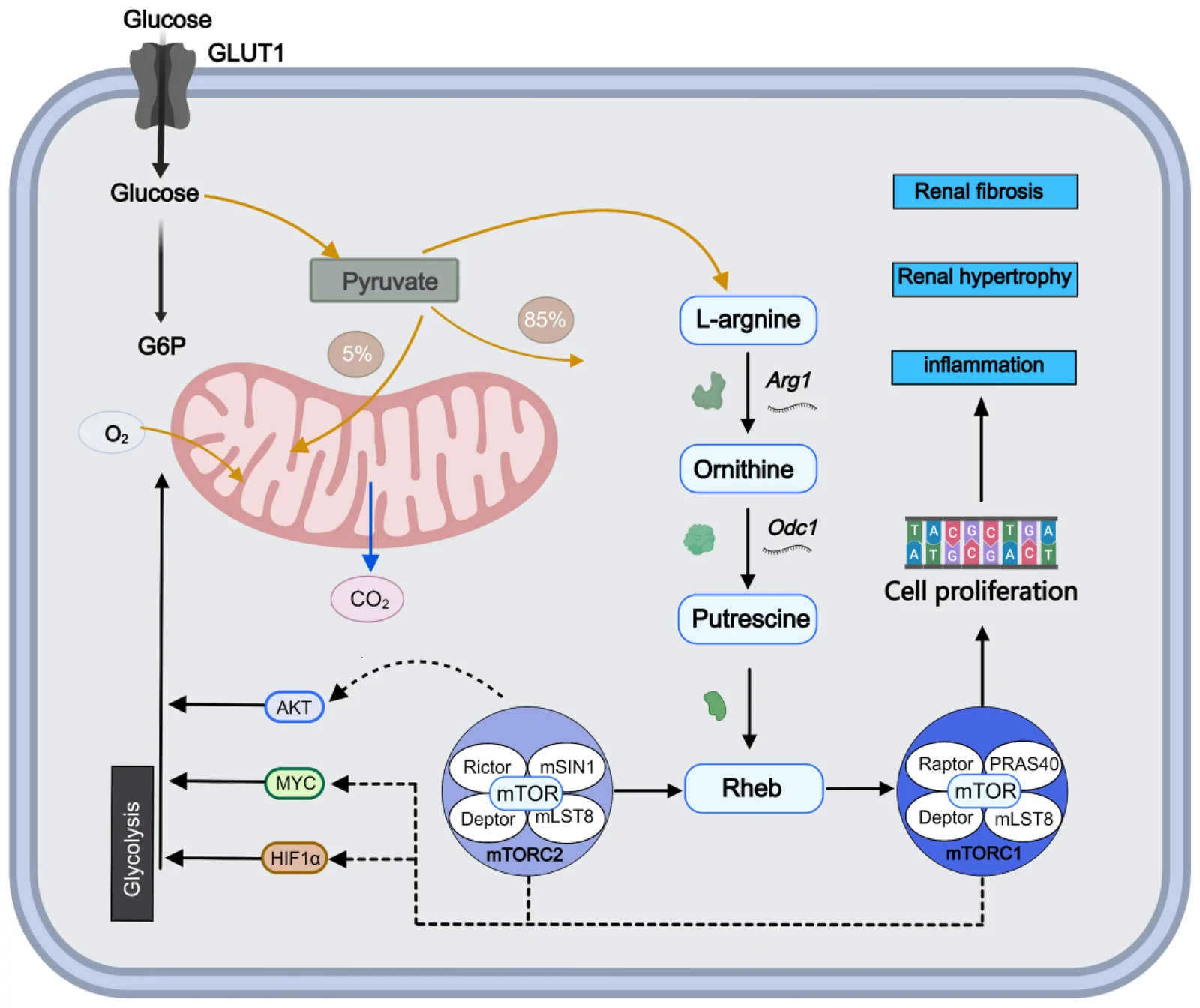
Pathological mechanism of ornithine in DKD. Under impaired glycolysis, ornithine metabolism is enhanced. Arginase 1 (ARG1) converts L-arginine to ornithine, which is then converted to putrescine by ornithine decarboxylase 1 (ODC1). The mTORC2 regulates AKT, mTORC1 drives downstream signaling of MYC and HIF1α, and regulates aerobic glycolysis. The mTOR pathway sensor Rheb exhibited enhanced activation upon putrescine stimulation. Activation of mTOR signaling leads to cytoskeleton remodeling of podocytes, causing renal hypertrophy, renal fibrosis, and inflammation. This image was created with MedPeer (www.medpeer.cn).

Despite the numerous associations reported between metabolites and DKD, translating these findings into clinical practice remains limited. Standardization and validation are major challenges. Most reported biomarkers come from single-center studies with small sample sizes and have rarely been validated in independent cohorts. The metabolite panels listed in **Table 1** show considerable heterogeneity, reflecting differences in study design, analytical platforms, sample types, and population characteristics. Large-scale, prospective validation studies using standardized protocols are urgently needed. Most candidate biomarkers lack quantitative assays suitable for clinical laboratories. Although research laboratories use LC-MS and NMR, which offer high analytical performance, these platforms are not widely applied in clinical settings. Developing robust, automated assays is essential for broad biomarker application. Therefore, combining metabolites with clinical parameters may be more effective than using either alone. Panels combining multiple metabolites, or metabolites with clinical variables, may achieve the performance required for clinical use. Machine learning approaches that integrate multi-metabolite signatures with clinical data are beginning to show promise (156, 157). At the same time, the translatability of animal models requires attention. **Table 2** summarizes metabolite alterations in animal models of DKD, revealing overlaps and differences compared with human studies. Although animal models recapitulate core metabolic pathways including amino acid metabolism, lipid metabolism, and the TCA cycle, model-specific changes also exist. Identifying which metabolic alterations are shared across species and which are model-specific is critical for using animal models to validate biomarkers and test mechanism-based therapies.

## 5 Targeting the metabolic signaling network as a therapeutic approach

Metabolites can act as signaling molecules that activate receptors (such as the AGEs–RAGE signaling axis), serve as substrates for epigenetic modifications, and function as mediators of intercellular communication. Metabolic dysregulation is not a late consequence of kidney injury; it occurs before clinical deterioration and acts as an early driver of that deterioration. This proposed causal role, supported by mechanistic evidence from experimental models, makes metabolites and their metabolic pathways promising therapeutic targets. The metabolic alterations in DKD are not isolated defects. Effective therapy may therefore require multi-pronged strategies—either by intervening on multiple nodes simultaneously or by targeting master regulators that control entire pathway modules. In this section, we classify emerging therapeutic strategies into four levels based on the sequential stages of a metabolite from production to elimination in the body (source intervention, signal blockade, metabolite correction, and global modulation). This framework is intended to guide the rational selection and combination of therapies to achieve maximal efficacy. The therapeutic approaches discussed below are at various stages of development. Readers should note that while some interventions are part of standard clinical care, others are strictly preclinical or exploratory. Their clinical and preclinical evidence is clearly distinguished in the subsequent sections.

### 5.1 Source intervention

#### 5.1.1 Targeting the gut microbiota

The gut microbiota is a major source of metabolites that enter the circulation and accumulate in DKD, including phenyl sulfate, indoxyl sulfate, and p-cresyl sulfate (185–187). Dysbiosis, characterized by reduced abundance of beneficial bacteria and increased opportunistic pathogens, exacerbates the production of these toxins while reducing the production of protective metabolites such as short-chain fatty acids (SCFAs) (188–190) (**Figure 6**). Several strategies are available to reshape the microbiota. Probiotics and prebiotics represent one such strategy. Administration of live beneficial bacteria or substrates that promote their growth can shift the microbial composition toward a healthier profile. Clinical studies have shown that probiotics reduce uremic toxin levels and alleviate kidney injury (191–193). Dietary fiber is another strategy. Fermentable fibers selectively promote the growth of SCFA-producing bacteria while suppressing toxin-producing microbes (194). Emerging evidence also supports fecal microbiota transplantation (FMT). While direct evidence in DKD is limited, transferring microbiota from healthy donors has been shown to restore metabolic homeostasis in other non-diabetic renal etiologies, such as refractory IgA nephropathy (195, 196), suggesting its potential translational value for DKD.

**Figure 6:**
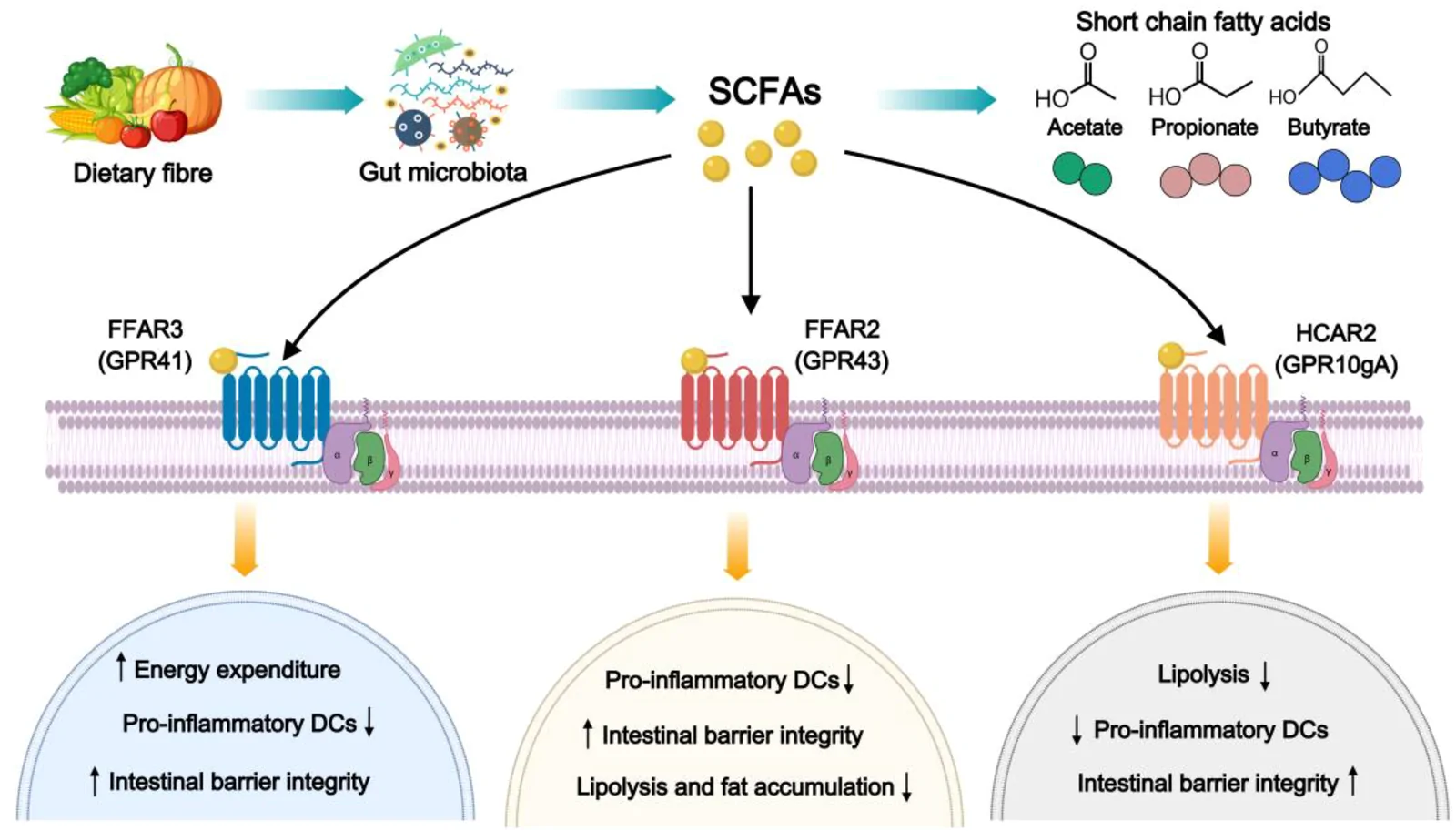
Role of SCFAs in DKD. The gut microbiota generates SCFAs through the fermentation of dietary carbohydrates. Acting as signaling molecules, SCFAs specifically activate three membrane receptors: FFAR3, FFAR2, and HCAR2. Activation of these receptors mediates multiple physiological effects in DKD, including: exerting anti-inflammatory actions and modulating immune homeostasis; maintaining intestinal barrier integrity; and suppressing lipid accumulation. This image was created with MedPeer (www.medpeer.cn).

#### 5.1.2 Targeting host metabolic enzymes

Another source intervention strategy is to target host enzymes that synthesize pathogenic metabolites. Preclinical studies have shown that sphingomyelin synthase inhibitors reduce ceramide production and ameliorate podocyte injury and glomerulosclerosis in DKD models (197). Thromboxane synthase inhibitors modulate renal prostaglandin metabolism and reduce albuminuria in patients with DKD (198). This approach targets the arachidonic acid pathway, linking lipid metabolism to hemodynamic and inflammatory effects. Furthermore, preclinical models have demonstrated that a specific inhibitor of adenine production attenuates renal hypertrophy and kidney injury in mouse models of DKD (199). Inhibitors of ornithine decarboxylase, which target the ornithine catabolic pathway, may prevent the cytoskeletal remodeling and foot process effacement driven by metabolites in this pathway. Specific inhibition of ODC1, the enzyme that converts ornithine to putrescine, has shown promise in preclinical models (152). The advantage of targeting host enzymes is that conventional drug development approaches can be used. The challenge is to achieve specificity without disrupting essential metabolic functions in other tissues.

### 5.2 Signal blockade

#### 5.2.1 Receptor modulators

RAGE antagonists represent a rational approach toblock the AGEs–RAGE axis. Soluble RAGE acts as a decoy receptor by binding AGEs and preventing receptor activation, and has shown renoprotective effects in preclinical studies (7, 200). Small-molecule RAGE antagonists are also under development, but none have yet been approved for DKD. Aryl hydrocarbon receptor (AhR) antagonists block the effects of tryptophan-derived metabolites such as Kyn. Kyn activation of AhR drives TGF-β1 expression and fibrosis (68, 201, 202). AhR antagonists have shown promise in preclinical studies of fibrotic diseases, but their application in DKD remains exploratory. Targeting G protein-coupled receptors (GPCRs) enables both blockade and activation strategies. Preclinical studies have shown that SCFAs activate GPR41 and GPR43, mediating anti-inflammatory effects (203, 204). Activation of these GPCRs reduces inflammation, regulates blood pressure, increases insulin sensitivity, and lowers oxidative stress and NF-κB signaling, thereby exerting protective effects in DKD (205).

#### 5.2.2 Signaling pathway inhibitors

Many metabolite-driven signals converge on intracellular pathways that can be targeted pharmacologically. In rodent models of DKD, mTORC1 is hyperactivated. mTOR inhibitors such as rapamycin suppress this hyperactivation, thereby restoring autophagy, reducing cell hypertrophy, and improving insulin sensitivity (206, 207). Genetic or pharmacological inhibition of TXNIP or the mTOR signaling pathway attenuates these pathological changes in DKD models (208). However, clinical translation of mTORC1 inhibitors has been limited by on-target toxicity. The first-in-human phase I trial of RMC-5552, a selective mTORC1 inhibitor, reported treatment-related adverse events including stomatitis (49% incidence), nausea (44%), and fatigue (42%) (209). Mechanism research shows that lipotoxicity, mitochondrial reactive oxygen species, oxidized lipids, and crystalline cholesterol converge on the NLRP3 signaling pathway in adipose tissue, myeloid cells, and the vascular compartment (210, 211). Preclinical studies have shown that small-molecule inhibitors targeting NLRP3—including those that modulate NLRP3 protein conformation, inhibit ASC oligomerization, block caspase-1 activity, or neutralize IL-1β—have therapeutic effects in kidney disease models. Several NLRP3 inhibitors are currently in clinical trials, raising the possibility that these agents may be used for DKD treatment (212, 213). Among NLRP3 inhibitors, AZD4144 (AstraZeneca) is currently the only agent in clinical trials for CKD (214). VTX2735 is being evaluated in phase 2 trials for recurrent pericarditis (215). NT-0796 is being evaluated in phase 2 cardiometabolic trials in patients with obesity and elevated cardiovascular risk. The challenge with pathway inhibitors is achieving tissue specificity while avoiding off-target effects. Nanoparticle formulations and EV-based delivery strategies may help overcome this limitation.

### 5.3 Metabolite correction

#### 5.3.1 SCFAs

SCFAs (acetate, propionate, and butyrate) are reduced in patients with DKD, and their decreased levels in feces and serum correlate with poorer kidney outcomes (216). Direct supplementation with SCFAs has demonstrated renoprotective effects in preclinical models. Butyrate ameliorates DKD-induced kidney injury by inhibiting NF-κB signaling via GPR43, reducing oxidative stress, and suppressing mesangial matrix accumulation (217). An inulin-type fructan-induced microbiota shift-characterized by enrichment of SCFA-producing genera like Akkermansia and Candidatus Saccharimonas—elevated acetate levels in feces and serum of db/db mouse models. This acetate mediated renal protection through improved glucose metabolism, reduced glycotoxicity, and enhanced mitochondrial function (218). Administration of acetate in mouse models recapitulated the benefits of fiber supplementation (219). Clinical translation of SCFAs faces preparation challenges; these volatile fatty acids have poor pharmacokinetic properties and require appropriate delivery strategies to achieve therapeutic concentrations.

#### 5.3.2 Amino acids and intermediary metabolites

Preclinical studies have shown that glycine supplementation reduces apoptosis in human proximal tubular cells by modulating the AMPK/mTOR pathway (172, 220, 221). Given its safety profile, glycine is an attractive candidate for clinical testing. β-Hydroxybutyrate (BHB), a ketone body produced during exercise or fasting, inhibits NLRP3 inflammasome activation and reduces inflammation (222, 223). Ketone ester supplements are being developed for various indications and could be tested in DKD. Ursodeoxycholic acid is a secondary bile acid that can reduce renal cell death induced by mild endoplasmic reticulum stress in DKD mice and upregulate Bcl-2 expression (224, 225). It is already approved for cholestatic liver disease, facilitating repurposing for DKD. Metabolite supplementation is safe, but if metabolite depletion is secondary to an ongoing pathogenic process rather than a primary deficiency, the efficacy of supplementation may be limited.

### 5.4 Global modulation

#### 5.4.1 Exercise

Preclinical studies have shown that exercise training exerts renoprotective effects through multiple mechanisms involving small-molecule metabolites (226, 227). Exercise improves mitochondrial dynamics in skeletal muscle and kidney, enhances oxidative capacity, and reduces ROS production (228–230). Clinical research has found that exercise-induced alterations in the gut microbiota correlated closely with improvements in glucose homeostasis and insulin sensitivity (231). Exercise also elevates glutathione levels and reduces oxidative stress markers (232, 233). The pleiotropic effects of exercise make it a powerful intervention, but patient adherence is challenging.

#### 5.4.2 Dietary intervention

Diet is the most readily modifiable factor influencing host metabolism and gut microbiota composition (234–237). Dietary fiber has shown consistent renoprotective effects. In a mouse model of type 2 diabetes, inulin supplementation increased SCFAs concentrations, reduced AGEs levels, and modulated multiple metabolic pathways, including glycerophospholipid metabolism, taurine metabolism, and tryptophan metabolism (238). In parallel, higher fiber intake is associated with reduced inflammation and mortality, particularly in patients with chronic kidney disease (239, 240). Different types of fiber may act through distinct mechanisms. Fermentable fibers act mainly through SCFA production, whereas insoluble fibers influence intestinal motility and toxin excretion via physical mechanisms (241, 242). Of note, individual responses to fiber may vary depending on baseline gut microbiota composition, suggesting that personalized dietary recommendations could optimize benefits (243–245). Polysaccharides from natural sources represent another dietary strategy. Plant-derived polysaccharides, such as those from corn silk, Chinese yam, red kidney bean, and small black bean, have shown renoprotective effects in preclinical studies. Their mechanisms include modulation of the gut microbiota, SCFA production, regulation of bile acid metabolism, and direct effects on renal metabolic pathways (246–249). For example, corn silk polysaccharide alleviates renal inflammation by restoring gut ecology and modulating uremic toxin metabolism (250). Chinese yam polysaccharide delays renal interstitial fibrosis by inhibiting glycerophospholipid metabolism through regulation of Gpd2 and Gpam (251).

The treatment of DKD is shifting from a “single-target” to a “network-based” strategy. Source intervention reduces the production of pathogenic metabolites, provided that the source is modifiable and the metabolites are truly pathogenic. Signal blockade acts downstream but carries a risk of compensation. Metabolite correction is simple and safe, but requires differentiation of whether a deficiency is a cause or a consequence. Global modulation offers broad benefits, but its mechanisms are complex. The optimal strategy often involves combining multiple levels—for example, dietary fiber together with SCFAs and exercise. Clinical management of patients can be guided by different metabolite profiles.

## 6 Toward metabolic precision medicine in DKD

### 6.1 From static visualization to dynamic prediction

In summary, multidimensional metabolomics has markedly advanced our ability to dissect the metabolic network in DKD. Spatial metabolomics has been validated for localizing metabolic injury within kidney tissue, but its application remains largely confined to research settings (252, 253). The next milestone is clinical spatial metabolomics. This would involve applying MSI to diagnostic kidney biopsies to provide metabolic information independent of conventional histopathology (254, 255). A future kidney biopsy report would not only report glomerulosclerosis and interstitial fibrosis scores, but also map the accumulation of lipotoxic lipids in podocytes and the mesangium. It would also show the localization of AGEs in specific vascular segments and the spatial distribution of TCA cycle intermediates (116). This “metabolic histology” holds promise for guiding individualized therapy, particularly for patients who clinically require kidney biopsies. However, given that DKD is predominantly diagnosed via non-invasive markers and routine biopsies carry inherent risks of hemorrhage, the application of MSI should be explicitly justified. MSI could be applied to kidney tissue that has already been obtained for a clinically indicated biopsy, to research biopsies conducted within approved protocols, or to residual clinical specimens after diagnostic workup, with informed consent and institutional approval, rather than suggesting routine biopsies for standard DKD patients solely for metabolic profiling. A patient with predominant podocyte lipotoxicity might benefit from ceramide synthesis inhibitors, whereas a patient with diffuse tubular mitochondrial dysfunction might respond to ketone bodies or SCFAs. Detection throughput, quantification methods, and integration with existing pathological workflows remain major technical barriers, but the clinical value is sufficient to drive the field forward.

Static metabolomics can identify which metabolites are altered. MFA goes further to reveal why they are altered. MFA can distinguish whether an elevation in a given metabolite results from increased production or decreased consumption, and can identify pathway bottlenecks that represent optimal intervention targets (256). Expanding the application of MFA in DKD research is a future direction. Current challenges include the complexity of *in vivo* tracer experiments and the need for specialized computational modeling (257, 258). However, advances in high-throughput omics—such as simplified tracers and automated analysis platforms—are making MFA more accessible (259). Combining static metabolomics with flux analysis enables mechanism-driven patient stratification. For example, one could not only identify that a patient has elevated ceramide levels, but also determine whether the cause is overproduction (suggesting a synthesis inhibitor) or underutilization (suggesting a catabolism enhancer).Furthermore, single-cell metabolomics is bringing a revolutionary prospect. The kidney is composed of dozens of cell types, each with a unique metabolic profile (260, 261). Conventional bulk metabolomics masks cell type-specific changes (262, 263). Although single-cell metabolomics is still in its early stages and limited by sensitivity and throughput, emerging platforms are gradually achieving the required sensitivity. These platforms include capillary electrophoresis-mass spectrometry, nanostructure-initiator mass spectrometry, and imaging mass cytometry (264–266). Once this technology matures, it will precisely locate the cellular origins of metabolic dysfunction, thereby facilitating the development of cell-specific targeted therapies.

### 6.2 From multi-omics to actionable knowledge

The technologies described above generate vast and complex datasets. These require equally sophisticated computational methods to extract biological insights and clinical value. Metabolites do not act in isolation. They are produced by gene-encoded enzymes, regulated by transcription factors, and influenced by the gut microbiota. Understanding metabolic networks therefore requires integrating metabolomics with genomics, transcriptomics, proteomics, and metagenomics (267). The next phase is causal multi-omics integration. This means moving beyond simple correlations to infer causal relationships across different omics datasets (268, 269). Relevant approaches include Mendelian randomization, which uses genetic variants tightly associated with a metabolite of interest as instrumental variables to test whether that metabolite has a causal effect on DKD (127). Bayesian network inference can reconstruct regulatory networks from multi-omics data (270, 271). Longitudinal multi-omics tracks how metabolic changes evolve over time and in response to interventions (257, 272). Such causal inference is essential for distinguishing merely correlative biomarkers from actionable pathogenic drivers. At the same time, machine learning has shown promise in predicting DKD progression using metabolomics data (273). However, the “black box” nature of many algorithms limits their clinical application. Cross-scale multimodal data fusion has emerged as a promising new paradigm, with predictive performance superior to that of single-modality approaches (274, 275). Molecular omics signature-driven prediction models for DKD shift the focus from traditional clinical indicators to molecular-level biomarkers. Han et al. identified PRKAR2B and TGFBI as potential markers of glomerular injury. Furthermore, proteomics-based models have further facilitated non-invasive diagnosis of DKD (276). The convergence of artificial intelligence, multi-omics, and mechanism-based therapeutic strategies paves the way for precision nephrology.

### 6.3 Metabolic phenotyping-driven precision medicine

The convergence of advanced technologies and computational methods points to a new paradigm for DKD management: metabolic precision medicine based on patients’ metabolic profiles. Metabolic phenotyping refers to stratifying patients into distinct subgroups according to their metabolic profiles. Each subgroup shares common metabolic drivers, disease progression trajectories, and therapeutic vulnerabilities. This approach goes beyond traditional clinical stratification and captures the underlying metabolic heterogeneity of DKD (277). This line of thinking aligns with the trajectory of precision oncology (e.g., molecular subtyping of breast cancer to guide individualized therapy) (278). Based on the literature reviewed in this article, we propose a preliminary, hypothetical framework of metabolic subtypes in DKD (**Table 3**). Building on this, we propose a closed-loop intervention system for metabolic precision medicine in DKD. This system consists of four sequential steps. The first step is baseline metabolic subtyping, in which patients’ metabolic subtypes is defined using multidimensional metabolomics. The second step is mechanism-based intervention, selecting a treatment strategy according to the subtype. The third step is dynamic monitoring, tracking treatment responses through serial metabolite measurements. The fourth step is adaptive therapy, adjusting interventions based on the monitoring results. This system stands in stark contrast to the current “one-size-fits-all” management paradigm that relies on eGFR and proteinuria. It is crucial to recognize that these proposed metabolic subtypes may substantially overlap in real-world clinical settings. An individual patient may simultaneously exhibit features of multiple subtypes, such as proposed TMAO-associated features, AGE accumulation, lipotoxicity, and mitochondrial dysfunction. Currently, these metabolic subtypes have not been externally validated in independent cohorts. Therefore, this subtyping framework should be explicitly viewed as a hypothesis-generating model. Future studies must evaluate the longitudinal stability of these subtypes during disease progression and prospectively assess their associations with clinical kidney outcomes in large, independent cohorts to validate their predictive and therapeutic utility.

**Table 3:** Proposed metabolite-based metabolic subtyping framework for DKD.

Metabotype	Metabolic characteristics	Key Metabolites	Interventions
TMAO-driven	Elevated TMAO, often with gut dysbiosis	TMAO, choline, carnitine	Microbiota modulation (fiber, probiotics), targeted TMA lyase inhibitors
BCAAs/mTOR-driven	Elevated BCAAs, activated mTORC1	L-leucine, l-valine, l-isoleucine	Dietary protein restriction, mTORC1 inhibitors
Purine/mTOR-driven	Elevated endogenous adenine, activated mTORC1	Adenine	Reduction of endogenous adenine synthesis, purine metabolism inhibitors
Ornithine-driven	Impaired glycolysis, activated ornithine catabolism, increased polyamine flux	Ornithine, putrescine, spermine	ODC1 inhibitors, polyamine pathway modulation, Rheb-mTOR inhibitions
AGEs/RAGE-driven	Elevated AGEs, oxidative stress markers	Methylglyoxal, CML, CEL	RAGE antagonists, strict glycemic control
AhR/tryptophan-driven	Increased Kyn pathway flux, AhR activation	L-Kyn	AhR antagonists
Lipotoxic	Elevated ceramides, DAG, lipids	Ceramides, DAG, LPC	Ceramide synthesis inhibitors, PPAR agonists, lipid-lowering agents
SCFAs-deficient	Low SCFAs, often with reduced fiber-fermenting bacteria	Acetate, propionate, butyrate	Fiber supplementation, SCFAs replacement, prebiotics
Mitochondrial dysfunction	TCA cycle alterations, elevated succinate and citrate	Succinate, citrate, fumarate	Ketone bodies, mitochondrial-targeted antioxidants, exercise

This framework is inferred from the literature reviewed in this paper and needs to be verified in a prospective cohort.

## 7 Conclusions and outlook

The realization of this vision faces multiple challenges. At the technological level, omics assays need standardization. Rapid point-of-care metabolite detection methods must be developed. Spatial and single-cell metabolomics require rigorous validation using clinical samples. At the biological level, we need to determine the stability of metabolic subtypes during disease progression, distinguish pathogenic drivers from secondary metabolic adaptations, and identify optimal combination strategies that address network redundancy. At the clinical level, trials must be designed to test the efficacy of metabolic phenotyping-guided treatment. Decision support tools must be developed to translate complex metabolic data into treatment recommendations. Addressing these challenges will require collaborative efforts from basic scientists, clinicians, bioinformaticians, and patients.

Despite these difficulties, the evidence assembled in this review firmly establishes a central role for metabolites in DKD. They are no longer viewed merely as passive byproducts of metabolism, but as active mediators that may drive disease pathogenesis, supported by associative clinical evidence and mechanistic experimental data. We advocate a new metabolite-centric paradigm: understanding DKD as an integrated disorder of metabolic signaling networks rather than as abnormalities in isolated pathways. In this framework, metabolites act as receptor ligands, epigenetic modifiers, and intercellular messengers, weaving an interconnected network of injury signals. Multidimensional metabolomics, integrating static profiling, dynamic flux analysis, and spatial imaging, enables us to decode this network with unprecedented resolution. Biomarkers can be classified according to their clinical function into early detection markers, progression prediction markers, and pathogenic driver markers. Therapeutic interventions can be stratified into source reduction, signal blockade, metabolite correction, and global modulation. The convergence of these advances points to a transformative clinical paradigm: metabolic precision medicine in DKD. This paradigm assigns patients to metabolic subtypes based on their dominant metabolic drivers, matches them with corresponding mechanism-guided interventions, and evaluates treatment responses through dynamic monitoring of circulating metabolites, thereby achieving better outcomes. Small-molecule metabolites, once viewed as metabolic waste, have now become the key to understanding, diagnosing, and ultimately defeating DKD.
